# Comparative Genomics Identifies Features Associated with Methicillin-Resistant Staphylococcus aureus (MRSA) Transmission in Hospital Settings

**DOI:** 10.1128/msphere.00116-22

**Published:** 2022-05-17

**Authors:** Timileyin Adediran, Stephanie Hitchcock, Lyndsay M. O’Hara, Jane M. Michalski, J. Kristie Johnson, David P. Calfee, Loren G. Miller, Tracy H. Hazen, Anthony D. Harris, David A. Rasko

**Affiliations:** a Institute for Genome Sciences, University of Maryland School of Medicine, Baltimore, Maryland, USA; b Department of Microbiology and Immunology, University of Maryland School of Medicine, Baltimore, Maryland, USA; c Department of Pathology, University of Maryland School of Medicine, Baltimore, Maryland, USA; d Division of Infectious Diseases, Weill Cornell Medicine, New York, USA; e Lundquist Institute for Biomedical Innovation at Harbor-UCLA Medical Center, Torrance, California, USA; f Department of Epidemiology and Public Health, University of Maryland School of Medicine, Baltimore, Maryland, USA; University of Michigan-Ann Arbor

**Keywords:** MRSA, *Staphylococcus aureus*, comparative studies, transmission

## Abstract

Methicillin-resistant Staphylococcus aureus (MRSA) is a serious public health concern in the United States. Patients colonized and/or infected can transmit MRSA to healthcare workers and subsequent patients However, the components of this transmission chain are just becoming evident, including certain patient factors, specific patient-healthcare worker interactions, and microbial factors. We conducted a comparative genomic analysis of 388 isolates from four hospitals in three states: Maryland, California, and New York. Isolates from nasal surveillance or clinical cultures were categorized as high, moderate, or low transmission surrogate outcomes based on the number of times the species was identified on the gloves or gowns of healthcare providers. The comparative analyses included a single gene, multigene, and core genome phylogenetic analysis, as well as a genome-wide association analysis to identify molecular signatures associated with the observed transmission surrogate outcomes, geographic origin, or sample source of isolation. Based on the phylogenetic analysis, 95% (*n* = 372) of the MRSA isolates were from four well-described genomic clades, with most of the isolates being part of the USA300 containing clade (*n* = 187; 48%). Genome-wide association studies also identified genes that were exclusive or prevalent among specific geographic locations. The identified genes provide insights into the transmission dynamics of MRSA isolates providing additional insights into the basis of the geographical differences of MRSA for molecular diagnostics.

**IMPORTANCE** Methicillin-resistant Staphylococcus aureus (MRSA) is considered a serious threat to public health and contributes to the dissemination of S. aureus in both the healthcare and community setting. Transmission of MRSA between patients via healthcare worker (HCW) has been described. However, what is not understood are the genetic determinants that contribute to the transmission of MRSA from patients to HCWs. In this study, we demonstrated that certain genes may be associated with transmission in the hospital setting.

## INTRODUCTION

Methicillin-resistant Staphylococcus aureus (MRSA) is a common cause of healthcare-associated infections (HAIs) and community-associated infections in the United States and is the leading cause of mortality among patients who develop HAIs (1, 2). MRSA can cause a wide range of illnesses, including soft tissue infections, pneumonia, and sepsis, and occurs frequently during hospitalization (10.1 discharges per 1,000 hospitalizations) (3). A third of all individuals in the United States are colonized with S. aureus, and 2% are colonized with MRSA (4, 5). Patients who are colonized with MRSA can become a reservoir of transmission and have the potential to spread this bacterium to susceptible HCW and other patients in a hospital setting (6). These transmission events may result in the development of symptomatic infection or asymptomatic colonization among patients who acquire MRSA (7).

The likelihood of transmission of a bacterial isolate from a patient to an HCW can be increased by several factors, including patient characteristics and healthcare worker (HCW)-patient interaction factors (8, 9). Patient-level risk factors of MRSA colonization and/or infection among patients hospitalized in the intensive care unit (ICU) include medical comorbidities and the presence of medical devices (10–13). Moreover, several recent studies have examined HCW-patient interaction factors that facilitate bacterial transmission (8, 9, 14, 15). These studies determined that HCW gowns or gloves are contaminated about 16% of the time after any patient contact. O’Hara et al. (9) identified certain HCW activities, such as wound care (OR: 1.57, CI: [1.57, 2.46]), bathing/hygiene (OR: 1.69, CI: [1.30, 2.19]), and manipulation of IV/tubing (OR: 1.22, CI: [1.04, 1.43]), that resulted in increased transmission of MRSA from the patient to an HCW gown and gloves. Additionally, certain HCW categories, such as occupational/physical therapists (OR: 6.96, CI: [3.51, 13.79]), respiratory therapists (OR: 5.34, CI: [3.04, 9.39]), and nurses (OR: 3.09, CI: [1.84, 5.19]) represented the greatest risk for glove and gown contamination likely due to the activities that they are performing on colonized or infected patients (9).

The genetic determinants of virulence for MRSA have been explored in detail. However, it is unclear if certain genetic virulence determinants may contribute to the altered transmission of MRSA (6, 16). Many adhesive mechanisms of MRSA involve the attachment of MRSA to host cells and to artificial surfaces (e.g., gloves and gowns of healthcare workers) including microbial surface components that recognize adhesive matrix molecules (MSCRAMMs), as well as the synthesis of polysaccharides and intercellular adhesion proteins, which were identified via traditional methods of identification (17–20). The *sasC* genes have been known to encode colonization factors that promote the spread of MRSA through microbial cell aggregation and biofilm formation (21, 22). However, it is not known whether these virulence factors contribute to the identified glove and gown transmission surrogate outcome.

In a previous study from our group, we identified that we could categorize MRSA isolates into glove and gown transmission surrogate outcomes based on the frequent or infrequent transmission to the HCW gowns and gloves, using a culture-based technique (9). In the current study, as in the previous study by O’Hara et al. (9), bacterial transmission is measured by the frequency of HCW glove or gown contamination after performing patient care activities among patients who were colonized and/or infected with MRSA. The objective of the current study was to use comparative genomics to identify genomic regions and genes that are associated with isolates that display the high or low transmission surrogate outcomes and to characterize MRSA genomic content among a large, multistate cohort. We hypothesized that adhesive mechanisms, biofilm aggregation, regulation of virulence, antibiotic resistance mechanisms, and mechanisms of persistence may all play a role in transmission either directly or indirectly. Here, we identified genetic characteristics, such as the LPGTX-motif encoding gene and *sasG* intercellular aggregation genes, which are both adhesive proteins that may contribute to geographical differences in the hospital setting. Overall, this study provides insight into the genetic components of MRSA transmission.

## RESULTS

### Phylogenetic analysis of MRSA strains.

**FIG 1:**
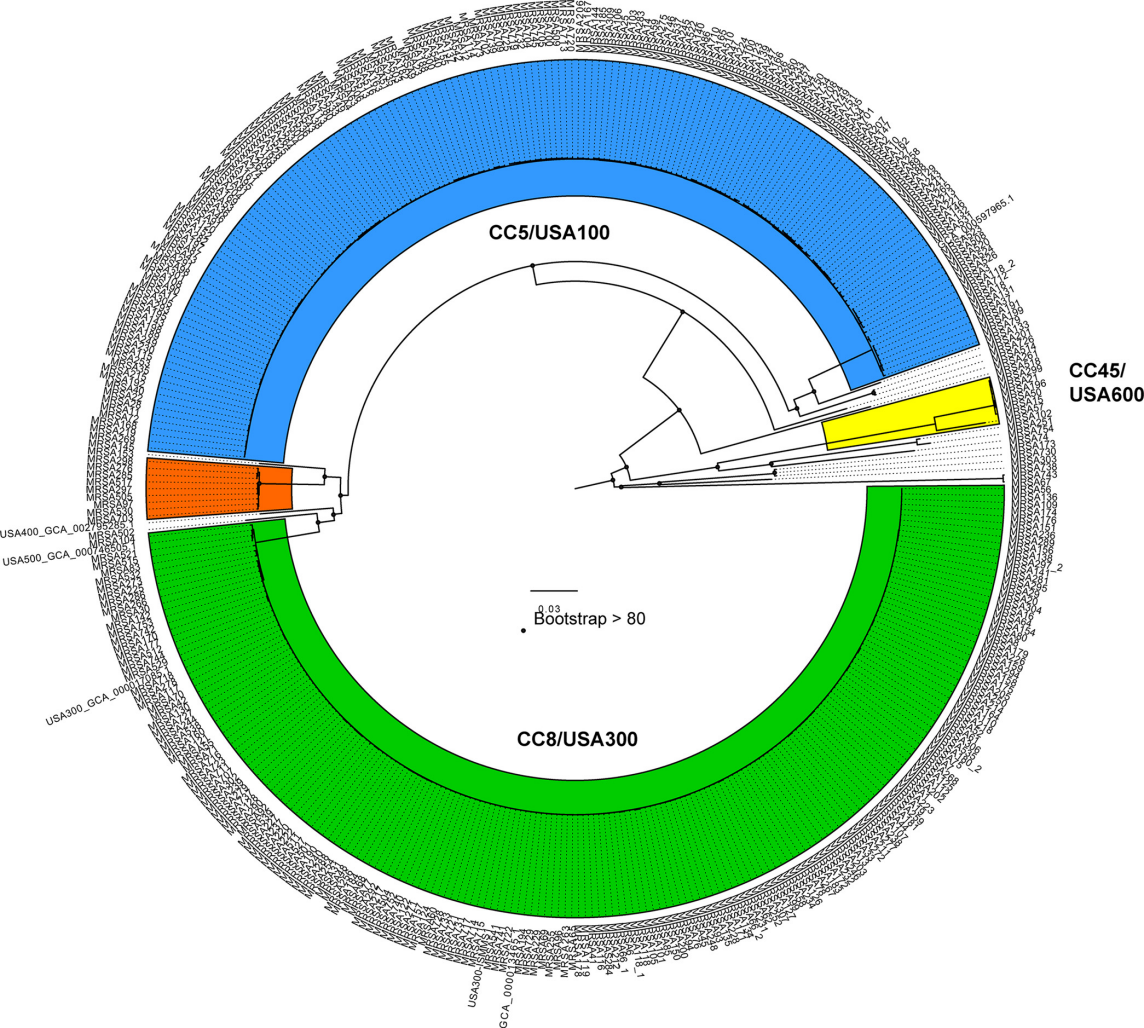
Phylogenetic analysis of 388 newly sequenced MRSA isolates and several previously sequenced MRSA isolates. Genomes were aligned to the S. aureus USA300-ISMMS1 chromosome (GenBank accession number NZ_CP007176) and a total of 157978 SNPs were identified using ISG.26RAxML (60) was used to create the phylogenetic tree using 100 bootstrap replicates and FigTree was used for visualizations (23, 24). The isolates highlighted in green are clade 1, those in blue are clade 2, those in orange are clade 3, and those in yellow are clade 4. Other isolates not in the four major clades are uncolored. The black dots at the nodes indicate greater than 80% bootstrap support.

Phylogenomic analysis was performed on the 388 MRSA isolates (Table 1, Table S1) and six additional reference genomes that were publicly available in GenBank (23, 24). There were four main genomic clades identified that represent over 95% of the isolates examined in this collection of isolates (Fig. 1 and Table S1). These major clades were aligned with the three dominant MLST clonal complexes (Table S1). Of the four genomic clades, 48.2% (*n* = 187) of the isolates were in clade 1 (USA300), 43.6% (*n* = 169) of the isolates were in clade 2 (USA100), 2.3% (*n* = 9) of the isolates were in clade 3 (USA500), and 1.8% (*n* = 7) in clade 4 (USA600). The remaining 16 isolates (4.1%) could not be assigned to any of these genomic clades.

**TABLE 1:** Summary of isolates examined in this study (*n* = 388)

		Culture type		Transmission status^*a*^		
Location	No. of isolates	Clinical cultures	Surveillance cultures	High	Moderate	Low
Maryland	295	115	180	35	123	137
New York	42	42	0	10	13	19
California	51	30	21	8	16	27

a MRSA isolates were classified by three different surrogate outcomes: (i) high transmitters, isolates that were identified on the gloves and gowns of HCW for more than 50% of HCW-patient interactions; (ii) mid transmitters, isolates that were identified on the gown and gloves for less than 50% of HCW-patient interactions; (iii) low transmitters, no transmission event occurred.

10.1128/msphere.00116-22.2 TABLE S1 Isolate metadata Download Table S1, XLSX file, 0.1 MB. Copyright © 2022 Adediran et al. 2022 Adediran et al. https://creativecommons.org/licenses/by/4.0/ This content is distributed under the terms of the Creative Commons Attribution 4.0 International license.

We divided the isolates based on transmission frequency: high, moderate, and low transmitters based on previously identified criteria. However, for these analyses, we focused on the high and low transmitter surrogate outcome groups (9). There were no differences in the number of isolates within each of the genomic clades among the transmission groups (*P* = 0.31) (Table 2). Among the high transmitter isolates, 52.8% (*n* = 28) were in genomic clade 2, while 50.8% (*n* = 21) of low transmitters were in genomic clade 1. Similarly, there were no differences in the distribution of the isolates in the phylogeny among surveillance/clinical cultures or body site of isolation (*P* = 0.29 and *P* = 0.46, respectively) (Table 2). However, there is a statistical difference in phylogeny groups among geographic locations (*P* = 0.03) (Table 2). We identified an association with the phylogenomic groups among MD and NY isolates (*P* = 0.02). Most NY isolates (54.6%) and CA isolates (45.1%) were in genomic clade 2, whereas the MD isolates were more likely to be from genomic clade 1 (51.5%).

**TABLE 2:** Distribution of MRSA isolates in clades (*n* = 388)

Location	Clade 1 *n* (%)	Clade 2 *n* (%)	Clade 3 *n* (%)	Clade 4 *n* (%)	Other *n* (%)	*P* value
Maryland	152 (80.9)	123 (72.8)	5 (55.6)	7 (100.0)	8 (50.0)	
New York	13 (6.9)	23 (13.6)	3 (33.3)	0 (0.0)	3 (18.8)	
California	22 (11.7)	23 (13.6)	1 (11.1)	0 (0.0)	5 (31.2)	
Clinical presentation						*P* = 0.29^*a*^
Clinical	84 (44.9)	84 (49.7)	7 (77.8)	2 (28.6)	10 (62.5)	
Surveillance	103 (55.1)	85 (50.3)	2 (22.2)	5 (71.4)	6 (37.5)	
Transmission surrogate outcome						*P* = 0.31^*a*^
High	21 (11.2)	28 (16.6)	0 (0.0)	2 (28.6)	2 (12.5)	
Moderate	73 (39.0)	70 (41.4)	3 (33.3)	0 (0.0)	6 (37.5)	
Low	93 (49.7)	71 (42.0)	6 (66.7)	5 (71.4)	8 (50.0)	

a Calculated using $\chi^{2}$.

### Clinical characteristics and transmission.

We examined different clinical characteristics of the hospitalized patients based on the transmission types of the isolates. We determined there was an association between patients with a peripherally inserted central catheter (PICC) and transmission surrogate outcomes (Table 3). Based on this observation, we analyzed if there were genes associated with PICC lines. There were no genes exclusive to the patients with a PICC line compared to patients with no PICC line. However, 22 genes were more prevalent among patients with a PICC line compared to patients without a PICC line (*P* < 0.05). Genes that were more prevalent in this group included LPXTG-motif encoding genes and *sas*C genes (Table S5).

**TABLE 3:** Clinical characteristics of patients by transmission surrogate outcomes

	High (*n* = 53)	Mid (*n* = 150)	Low (*n* = 181)	*P* value^*a*^
Artificial airway or ET tube	29 (54.6)	91 (60.1)	101 (55.8)	0.61
Central line or PICC line	24 (45.3)	78 (52.0)	115 (63.5)	<0.001
Chest tube	4 (7.6)	8 (5.3)	19 (105)	0.23
Diarrhea	11 (20.8)	43 (28.7)	36 (19.9)	0.15
Foley	31 (58.5)	80 (53.3)	106 (58.6)	0.6
Nasogastric tube	30 (56.6)	84 (66.0)	99 (54.7)	0.96
Rectal tube	9 (17.0)	28 (18.7)	24 (13.3)	0.4
Surgical drain	9 (17.0)	29 (39.2)	36 (19.9)	0.89
Wound	30 (56.6)	89 (59.3)	109 (60.2)	0.89

a Calculated using $\chi^{2}$.

10.1128/msphere.00116-22.6 TABLE S5 Identification of genes in PICC and non-PICC line isolates Download Table S5, XLSX file, 0.01 MB. Copyright © 2022 Adediran et al. 2022 Adediran et al. https://creativecommons.org/licenses/by/4.0/ This content is distributed under the terms of the Creative Commons Attribution 4.0 International license.

### Molecular typing.

**(i) *spa*-typing.** The *spa*-typing technique is a single locus typing scheme that compares the 3′ coding region of S. aureus protein A and assigns a type (25). We assigned 72 *spa*-types to 382 isolates, with the remaining six isolates unable to be assigned a *spa*-type. This level of unassigned isolates is not uncommon in genomic studies (26). Among the isolates that could be assigned a *spa*-type the most common was t008 (*n* = 145/382; 37.9%), and the second most common *spa*-type was t002 (*n* = 84; 21.6%) However, there were no statistical differences between *spa*-type and transmission (*P* = 0.36) (Table 4).

**TABLE 4:** Molecular typing schema applied

*spa*-typing	n (%)	SCC*mec*	*n* (%)	MLST	*n* (%)
t008	145 (37.4)	IV	225 (58.0)	8 (CC8)	169 (43.6)
t002	84 (21.6)	II	134 (34.5)	5 (CC5)	77 (19.8)
t242	24 (6.2)	V	6 (1.5)	105 (CC5)	63 (16.2)
t105	12 (3.1)	VII	2 (0.52)	225 (CC5)	11 (2.8)
t045	9 (2.3)	No SCC*mec* type	22 (5.8)	72 (CC8)	8 (2.1)
t211	9 (2.3)			45 (CC45)	6 (1.5)
t024	7 (1.8)			840 (CC5)	6 (1.5)
t088	6 (1.5)			59	3 (0.77)
t1081	6 (1.5)			3390 (CC5)	3 (0.77)
t548	4 (1.0)			88, 152, 398	2 (0.52)
t121	3 (0.8)			80, 106, 121, 109 (CC1). 1252 (CC8), 1540 (CC8), 1750 (CC8), 22 (CC22), 2253 (CC8), 231 (CC5), 36 (CC30), 54 (CC45), 772 (CC1)	14 (3.6)
t062, t064, t068, t148, t1548, t216, t2308, t2703, t450, t535, t586, t723, t856	2 (0.01)			2253 (CC8)	
Other	47 (12.1)			ND^*a*^	23 (5.9)
No *spa*-type	7 (1.8)				

a ND, no MLST was determined.

**(ii) SCC*mec* typing.** The SCC*mec* typing method uses the mobile genetic element Staphylococcal cassette chromosome, *mecA* or *mec C* genes and assigns a SCC*mec* type (27). We identified four SCC*mec* types among 367 isolates. Twenty-one isolates were unable to be typed using this method. The most prominent type of SCC*mec* type among the isolate was type IV (*n* = 225; 61.3%) following type II (*n* = 134; 36.5%). SCC*mec* type IV was the most prominent type among the high (26/53; 49.1%) and low transmitter groups (113/183; 61.7%) (Table S1).

**(iii) Multilocus sequencing typing (MLST).** MLST is a typing method that uses the seven housekeeping genes found in all S. aureus to characterize the genomic divergence of isolates (28). A total of 25 MLST types were identified in this collection of isolates. However, 308/388 (79.4%) of the isolates were represented in three sequence types (ST8, ST5, and ST105). Additionally, there were 18 isolates (4.6%) that did not belong to any of the known ST groups (Table S1). These results highlight the clonal nature of the S. aureus isolates.

### Genome-wide association comparisons.

**(i) High versus low transmission surrogate outcome.** We identified that 53 isolates could be associated with the high transmission surrogate outcome and 183 isolates were associated with the low transmission surrogate outcome. The remaining 152 isolates were moderate-level transmitters and are not examined in detail in these analyses. We examined the 8,768 potential coding sequences from the 236 isolates in the high and low transmission groups to determine which genes were associated with the MRSA isolates from the high or low transmission groups. Among these genes, there were 4,606 genes (52.5%) that were shared among all the isolates indicating a high level of clonality.

Three genes were exclusively identified among the low transmission group isolates. These exclusively low transmission associated genes all encoded hypothetical proteins. Additionally, we identified four genes that were exclusive to the high transmission group, encoding two hypothetical proteins, one surface G protein (SasG) (29, 30), and one tandem five-TM protein. Of note, SasG is an immunodominant-containing protein that aids in the promotion of biofilm formation (29, 30). While these genes are exclusive in this collection of isolates, they are not statistically significant in the greater comparison of all genes and isolates due to the presence of the genes in relatively few high or low transmission isolates, respectively. When the analysis was corrected for multiple observations (Bonferroni correction [*P* < 0.05]) there were no genes that were significantly more prevalent in each of the groups.

**(ii) Source of isolates.** We further analyzed the genomic content of the isolates based on the source of culture because certain genes may play a significant role in a certain body location. Most of the isolates were from nasal surveillance specimens (*n* = 201/388; 51.8%) (Table S1). The remaining isolates were from clinical cultures (*n* = 187/388; 48.2%). The breakdown of the clinical isolates was as follows: 99 (52.9%) were from sputum, 35 (18.7%) from blood, 9 (4.8%) from wounds, 6 (3.2%) from urine, and 38 (20.3%) from other body sites such as tissue biopsy specimens. For this analysis, we combined all the clinical sites (*n* = 88) except sputum (*n* = 99) and compared these clinical sites to the isolates from nares surveillance cultures (*n* = 201). We examined sputum cultures separately from the other clinical sites because we believed that the gene hits would be different in comparison to other clinical culture sites. Comparison of genomes from nares isolates to genomes from all other body sites isolates identified no genes to be more prevalent among one of these groups (Bonferroni correction [*P* < 0.05]). Similarly, there were no genes that were identified to be more prevalent among clinical isolates compared to the genomes from the surveillance isolates.

**(iii) Geographic location.** Isolates were sampled at four hospitals in three states: Maryland, California, and New York (Table 1). Most of the isolates were from Maryland (*n* = 295; 76.0%). A comparison of Maryland MRSA isolates to the other geographic isolates determined 29 prevalent genes based on the Bonferroni correction (*P* < 0.05). Among these prevalent genes from the Maryland isolates, we identified 43 genes that were exclusive to Maryland isolates from Bonferroni correction. These genes include the LPXTG-motif encoding gene and surface G protein (SasG) (Table S2). LPXTG is a conserved peptide motif that is thought to contribute to the surface protein's ability to anchor onto the cell wall, thus also potentially enhancing adhesion and transmissibility among these isolates (31–33). Of note, SasG is an immunodominant protein that aids in the promotion of biofilm formation (29, 30).

10.1128/msphere.00116-22.3 TABLE S2 Genes associated with a geographical area of isolates Download Table S2, XLSX file, 0.1 MB. Copyright © 2022 Adediran et al. 2022 Adediran et al. https://creativecommons.org/licenses/by/4.0/ This content is distributed under the terms of the Creative Commons Attribution 4.0 International license.

Among isolates from the NY hospital, there were nine prevalent genes (Table S2). In contrast, 54 genes were statistically significant using a Bonferroni correction among the isolates from California (*P* < 0.05). There were zero exclusive genes among the California isolates (Table S2). The prevalent genes found in the California isolates include but are not limited to three bacitracin resistance proteins (*bacA*), two transposase IS66 family proteins, and several hypothetical proteins. BacA is a protein that encodes resistance to the antibiotic bacitracin, which results in the inhibition of the cell wall biosynthesis (34, 35). In addition to this resistance mechanism prevalent among the California isolates, transposase IS66 family protein is a protein that is required for transposition (36).

**(iv) Prevalence of identified virulence factors in MRSA genomes.** There is a large body of literature on the virulence factors of S. aureus in general and MRSA specifically (6, 16, 37–39) (Table S3). Genes known for virulence identified among the 388 MRSA isolates include but are not limited to several enterotoxins, fibrinogen binding proteins, staphlyocoagulase, immunoglobulin G-binding protein A, and capsular polysaccharide biosynthesis proteins (6, 16, 37–41). Most of the virulence genes appear to be highly conserved among all the MRSA isolates in this analysis (Fig. S1). However, many of the enterotoxins (i.e., K, Q, E, C, and H) have divergent genes, which are involved in the mechanism of food poisoning (42). The exfoliative protein B gene appears to be divergent as well, which is known to be important in scalded skin syndrome (6, 16). Additionally, there was one virulence factor, fibronectin-binding protein A (*fnbA*), that was statistically more prevalent in low transmitters compared to high transmitters (*P* < 0.05) (Table S4). The *fnbA* encodes a protein known to be involved in biofilm formation (43). Overall, the traditional S. aureus virulence factors, as listed in Table S3 and S4, do not appear to be involved with the transmission surrogate outcomes observed in this study as they are overrepresented in each of the groups examined.

10.1128/msphere.00116-22.1 FIG S1 Heatmap of 236 high and low transmission MRSA isolates using LS-BSR analysis for known virulence factors for MRSA. LS-BSR analysis to detect if virulence factors were associated with the high/low transmission isolates. Each column represents an isolate and each row represents a predicted gene, the color in the plot indicates the level of similarity in each genome based on the LS-BSR values. Download FIG S1, TIF file, 10.7 MB. Copyright © 2022 Adediran et al. 2022 Adediran et al. https://creativecommons.org/licenses/by/4.0/ This content is distributed under the terms of the Creative Commons Attribution 4.0 International license.

10.1128/msphere.00116-22.4 TABLE S3 List of previously identified virulence factors of MRSA Download Table S3, XLSX file, 0.01 MB. Copyright © 2022 Adediran et al. 2022 Adediran et al. https://creativecommons.org/licenses/by/4.0/ This content is distributed under the terms of the Creative Commons Attribution 4.0 International license.

10.1128/msphere.00116-22.5 TABLE S4 Identification of virulence genes among high versus low transmitters Download Table S4, XLSX file, 0.01 MB. Copyright © 2022 Adediran et al. 2022 Adediran et al. https://creativecommons.org/licenses/by/4.0/ This content is distributed under the terms of the Creative Commons Attribution 4.0 International license.

## DISCUSSION

The objective of this study was to characterize the genomic content of MRSA clinical/surveillance isolates from a multistate cohort and specifically identify genetic determinants of transmission. There have been no studies to our knowledge that have characterized the genetic determinants of transmission among MRSA isolates. Our average predicted genome size was 2,857,897 with a range of 2,682,854 bp to 3,009,992 bp among 388 isolates, which is similar to other studies (44). Large scale BLAST score ratio (LS-BSR) analysis predicted 8,768 genomic coding regions across the 388 MRSA isolates, which differs from previous studies of smaller isolate samples and predictions of pan-genome size (44, 45). Our study allowed the comparison of the genomic content between high and low transmission MRSA isolates, location, and clinical and surveillance isolates. Based on this novel analysis, we identified genes that were exclusive to geographic locations. However, we did not identify genes that were exclusive or more prevalent among high versus low transmission groups, clinical versus surveillance groups, or clinical source of isolation groups after correcting for multiple corrections. This is interesting because it would suggest that the genomic content is not directly related to the transmission surrogate outcomes observed.

From the phylogenetic analysis, we determined there were four major phylogenomic clades within the sequenced MRSA isolates. As expected, we see that these dominant phylogenomic groups represent common S. aureus genomic clades (6, 16) (Fig. 1), which correspond to the USA100 (CC5), USA300 (CC8), USA400 (CC1), and USA600 (CC45) groups that are common in healthcare settings (6, 16). Additionally, USA300 was the most prevalent group among the isolates analyzed and this corresponds with the most frequent emergent MRSA strains in a healthcare setting in North America (6, 16, 46). However, we have also identified 16 isolates that are outside these dominant clades. Additionally, we identified that the *spa*-types t008 and t002 were the most prevalent spa-types among our isolates, which is similar to the prior literature that identified *spa*-types t008, t002, and t242 to be the most prevalent in the United States (47). We also found that *spa*-type was statistically different between geographical locations, with t008 (84%) and t002 (67%) being more prevalent in MD than in California and New York (*P* < 0.001).

The LS-BSR analysis identified genomic content that is unique or more prevalent among high versus low transmission groups, clinical versus surveillance isolates, as well as geographic location. A study by McClure et al. (44, 48) examined genetic content to determine virulence genes in M92, a strain of MRSA found in a nasal sample from one hospital in Calgary, Alberta, Canada. This analysis was based on only one reference strain of MRSA that identified 3,152 coding regions, which was fewer than what we found and may not be representative of our diverse multistate cohort. The differences seen between the two studies may be due to our isolates being from diverse geographical regions and multiple body sites.

When comparing the genomic content among high versus low transmitters using the GWAS approach, we identified zero genes that were more or less prevalent in either of the groups when correcting for multiple observations. Similarly, we were unable to identify any genes that were prevalent or exclusive among isolates based on the source of isolation. However, we observed geographical differences in the presence or absence of certain genes.

Our results suggest that MRSA gene content does not significantly differ among the isolates from the identified transmission surrogate outcomes. However, the transmission of MRSA from the patient to an HCW occurs 16.2% of the time suggesting that activities performed by the HCW may be the main factor in transmission (9). These results combined with patient level and HCW-patient interaction factors are the first step to providing further knowledge on which patients need to be placed on contact precautions and contribute valuable information to clinical care.

## MATERIALS AND METHODS

### Isolate selection.

MRSA isolates were obtained from patient samples collected as a part of a cohort study previously described (9). This cohort contained 403 isolates from 403 patients treated at four hospitals in the following states: Maryland, New York, and California. Swabs were cultured using local hospital laboratory protocol (9). The 403 isolates were cultured from either the following body sites/sources: anterior nasal samples or the following clinical sample types: wounds, sputum, urine, blood, and other body sites (Table S1). Nasal surveillance was performed for infection control purposes to identify colonized patients. Clinical samples were ordered by physicians based on clinical signs and symptoms. To assess for MRSA transmission, gowns and gloves of HCW who entered the hospital room and provided care to the 403 patients were sampled immediately before doffing to determine if there were MRSA transmission to the gown and/or gloves. Swabs were cultured onto a CHROMagar MRSA and incubated overnight. 10 HCW-patient interactions were observed per patient. Based on the gown and glove contamination data, we defined MRSA isolates as high transmitters if the gloves and gowns of HCW were contaminated with MRSA in more than 50% of HCW-patient interactions. Whereas isolates were classified as low transmitters if there were no identified transmission events (Table 1). Those for which transmission was detected in 1 to 49% of interactions were considered moderate transmitters but were not included in the detailed comparative analysis in this study. A total of 15 isolates were not included in the comparative genomic analysis because upon sequencing and quality control of the genome data, the genome was determined to not be S. aureus, did not assemble adequately based on the N50 sequencing metric, or the total genome length or GC% not being similar to S. aureus. Thus, there were a total of 388 MRSA genomes for comparative analysis in the current study, for which we previously described the sequencing and assembly (49). The study was approved by Institutional Review Boards at the University of Maryland, Baltimore (HP-000066759-16), Weill Cornell Medicine, New York (1610017615), and LaBioMed, California (042087).

### Genome sequencing.

Genomic DNA was isolated from cultures grown in Lysogeny Broth overnight. DNA was extracted in 96-well format from 100 μL of the sample using the MagAttract PowerMicrobiome DNA/RNA kit (Qiagen, Hilden, Germany) automated on a Hamilton Microlab STAR robotic platform. Bead disruption was conducted on a TissueLyser II (20 Hz for 20 min) instrument in a 96-deep well plate in the presence of 200 μL phenol-chloroform. Genomic DNA was eluted in 90 μL water after magnetic bead cleanup. The resulting genomic DNA was quantified by Pico Green. The sequencing libraries were generated with the KAPA HyperPrep kit (catalog number KK8504) and sequenced on the Illumina HiSeq4000 using a 150bpX2 paired-end kit. Raw sequencing reads were filtered to remove contaminating *phiX* reads using BBDuk of the BBTools software suite (sourceforge.net/projects/bbmap/). The raw reads were also filtered to remove contaminating Illumina adaptor sequences and quality trimmed using Trimmomatic v.0.36 (50). The resulting filtered reads were then assembled using SPAdes v3.13.0 (51). The resulting assemblies were then filtered to contain only contigs longer than 500 bp with a k-mer coverage ≥5×. Genomes containing greater than 500 contigs or an aberrant GC percentage were removed from further analysis. A total of 388 genomes were used in the comparative analysis and the relevant genomic details and clinical data are included in Table S1 and were briefly described in Adediran et al. (49).

### Comparative genomics.

**(i) Phylogenetic analysis *in silico*.** Genotyper was used to examine the whole-genome content (23, 24). We compared the 388 MRSA genomes from this study, as well as multiple reference genomes with accession numbers: CP012120, NZ_CP009423, NZ_GG697985, NZ_CP029474, NZ_CP019574.1, GCA_003991015.1, GCA_000746515.1, CP000730, NC_007795, GCF_002795295.1, PDEY0000000, NC_002758, NC_009641, NC_021554, NC_017347, and NC_007793 using the USA300 reference genome S. aureus USA300-ISMMS1 isolate as the reference. The phylogenetic tree was visualized using FigTree v.1.4.0. (http://tree.bio.ed.ac.uk/software/figtree/). The prevalence of isolates in the genomic clades was compared among transmission groups, geographic location, and source of isolate using Fisher’s exact test using SAS v.9.4 (Cary, NC).

**FIG 2:**
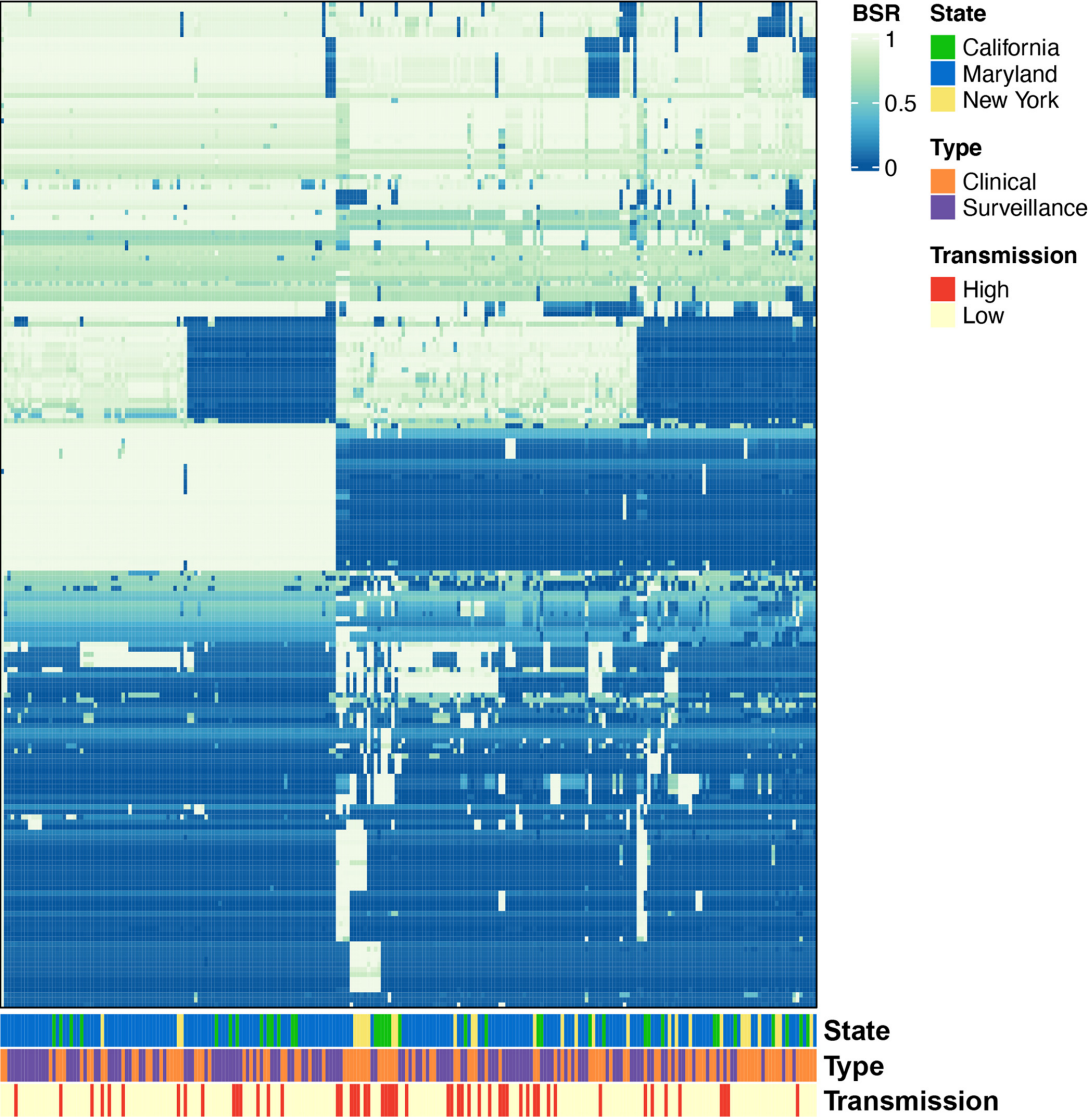
Heatmap of 236 high and low transmission MRSA isolates using LS-BSR analysis. LS-BSR analysis to detect if genomic content was associated with the isolates as clustered by presence and absence. Each column represents an isolate and each row represents a predicted gene, the color in the plot indicates the level of similarity in each genome based on the LS-BSR values which were generated previously. The heat map was visualized using the ComplexHeatmaps package of R v.4.0.2 (53, 54) generated previously. The heat map was visualized using the Heatmap 2 package of R v.4.0.2.

**(ii) Large scale BLAST score ratio (LS-BSR).** LS-BSR analysis of the complete genomic content was performed on the 388 isolates as previously described (49, 52). BSR values were visualized (Fig. 2) using the ComplexHeatmaps package of R v.4.0.2 (53, 54) to compare the presence or absence of genes to associate them with the observed transmission surrogate outcome (Data Set S1-Data Set S3). Hierarchical clustering of the rows and columns of the heat map was performed using default parameters. To determine if genes were present in greater frequency among transmission groups, phylogenetic groups, or clinical groups, we used Fisher's exact test to examine the frequency of these values. A *P* value less than 0.05 was considered significant for all comparisons. R version 4.02 was used for this analysis (54). A genome-wide association study was conducted among 388 MRSA isolates using Scoary v.1.6.16 (https://github.com/AdmiralenOla/Scoary) (55). Genes associated with the surrogate outcome of interest at *P* < 0.05 were considered significant with either Bonferroni or Benjamini-Hochberg correction. A comparison between surrogate outcomes of interest was conducted to determine if there were genes exclusive to one of the surrogate outcomes of interest.

10.1128/msphere.00116-22.7 DATA SET S1 LS-BSR matrix for comparison of MRSA isolates. Download Data Set S1, TXT file, 16.0 MB. Copyright © 2022 Adediran et al. 2022 Adediran et al. https://creativecommons.org/licenses/by/4.0/ This content is distributed under the terms of the Creative Commons Attribution 4.0 International license.

10.1128/msphere.00116-22.8 DATA SET S2 Generated FASTA from the LS-BSR analysis Download Data Set S2, TXT file, 7.9 MB. Copyright © 2022 Adediran et al. 2022 Adediran et al. https://creativecommons.org/licenses/by/4.0/ This content is distributed under the terms of the Creative Commons Attribution 4.0 International license.

10.1128/msphere.00116-22.9 DATA SET S3 Annotation for FASTA from the LS-BSR analysis Download Data Set S3, TXT file, 1.4 MB. Copyright © 2022 Adediran et al. 2022 Adediran et al. https://creativecommons.org/licenses/by/4.0/ This content is distributed under the terms of the Creative Commons Attribution 4.0 International license.

**(iii) Whole-genome MLST (wgMLST) analysis.** The seven genomically conserved housekeeping loci (*arcC*, *aroE*, *glpF*, *gmk*, *pta*, *tpi*, and *yqiL*) of the multilocus sequence typing (MLST) scheme previously developed were identified in each of the genomes (28). The allele numbers of each locus and the sequences types (STs) of each genome were determined using the BIGSdb software (https://pubmlst.org/saureus/) (Table S1) (56).

**(iv) *spa*-typing analysis.** *spa*-typing analysis was performed on the 388 MRSA isolates of interest using *spaT*yper 1.0 (Center for Genomic Epidemiology, Denmark, https://cge.cbs.dtu.dk/services/spatyper/). Genomes were uploaded to the website to identify the *spa* gene types for each of the individual isolates (26). Results for each isolate are presented in Table S1.

**(v) SCC*mec* typing analysis.** We conducted the SCC*mec* typing method on the 388 isolates of interest to determine the SCC*mec* element using SCCmec Finder1.2 (Center for Genomic Epidemiology, Denmark, https://cge.cbs.dtu.dk/services/SCCmecFinder) The 388 MRSA isolates were uploaded to determine *SCCmec* type. *SCC*mec types were computed based on the alignment and minimum length coverage of the alignment (57–59). MLST for each isolate is presented in Table S1.

### Data availability.

GenBank accession and short read archive numbers for these data are provided in Table S1.

## References

[1] Weiner LM, Webb AK, Limbago B, Dudeck MA, Patel J, Kallen AJ, Edwards JR, Sievert DM. 2016. Antimicrobial-resistant pathogens associated with healthcare-associated infections: summary of data reported to the national healthcare safety network at the centers for disease control and prevention, 2011–2014. Infect Control Hosp Epidemiol 37:1288–1301. doi:10.1017/ice.2016.174. 27573805 PMC6857725

[2] Moore C, Dhaliwal J, Tong A, Eden S, Wigston C, Willey B, McGeer A. 2008. Risk factors for methicillin-resistant staphylococcus aureus (mrsa) acquisition in roommate contacts of patients colonized or infected with MRSA in an acute-care hospital. Infect Control Hosp Epidemiol 29:600–606. doi:10.1086/588567. 18624667

[3] Klein EY, Mojica N, Jiang W, Cosgrove SE, Septimus E, Morgan DJ, Laxminarayan R. 2017. Trends in methicillin-resistant staphylococcus aureus hospitalizations in the United States, 2010–2014. Clin Infect Dis 65:1921–1923. doi:10.1093/cid/cix640. 29020322

[4] Gorwitz RJ, Kruszon‐Moran D, McAllister SK, McQuillan G, McDougal LK, Fosheim GE, Jensen BJ, Killgore G, Tenover FC, Kuehnert MJ. 2008. Changes in the prevalence of nasal colonization with Staphylococcus aureus in the United States, 2001–2004. J Infect Dis 197:1226–1234. doi:10.1086/533494. 18422434

[5] Centers for Disease Control U. Antibiotic resistance threats in the United States, 2019. www.cdc.gov/DrugResistance/Biggest-Threats.html. Accessed December 26, 2019.

[6] Turner NA, Sharma-Kuinkel BK, Maskarinec SA, Eichenberger EM, Shah PP, Carugati M, Holland TL, Fowler VG. 2019. Methicillin-resistant Staphylococcus aureus: an overview of basic and clinical research. Nat Rev Microbiol 17:203–218. doi:10.1038/s41579-018-0147-4. 30737488 PMC6939889

[7] Wertheim HFL, Melles DC, Vos MC, van Leeuwen W, van Belkum A, Verbrugh HA, Nouwen JL. 2005. The role of nasal carriage in Staphylococcus aureus infections. Lancet Infect Dis 5:751–762. doi:10.1016/S1473-3099(05)70295-4. 16310147

[8] Blanco N, O'Hara LM, Harris AD. 2019. Transmission pathways of multidrug-resistant organisms in the hospital setting: a scoping review. Infect Control Hosp Epidemiol 40:447–456. doi:10.1017/ice.2018.359. 30837029 PMC6897300

[9] O’Hara L, Calfee D, Miller L. 2019. Optimizing contact precautions to curb the spread of antibiotic-resistant bacteria in hospitals: a multicenter cohort study to identify patient characteristics and healthcare personnel interactions associated with transmission of MRSA. Clin Infect Dis 69:S171–S177. doi:10.1093/cid/ciz621. 31517979 PMC6761365

[10] McKinnell JA, Miller LG, Eells SJ, Cui E, Huang SS. 2013. A systematic literature review and meta-analysis of factors associated with methicillin-resistant Staphylococcus aureus colonization at time of hospital or intensive care unit admission. Infect Control Hosp Epidemiol 34:1077–1086. doi:10.1086/673157. 24018925 PMC3883507

[11] Khawcharoenporn T, Tice AD, Grandinetti A, Chow D. 2010. Risk factors for community-associated methicillin-resistant Staphylococcus aureus cellulitis–and the value of recognition. Hawaii Med J 69:232–236. 21229486 PMC3071185

[12] Hidron AI, Kourbatova EV, Halvosa JS, Terrell BJ, McDougal LK, Tenover FC, Blumberg HM, King MD. 2005. Risk factors for colonization with methicillin‐resistant Staphylococcus aureus (MRSA) in patients admitted to an urban hospital: emergence of community‐associated MRSA nasal carriage. Clin Infect Dis 41:159–166. doi:10.1086/430910. 15983910

[13] Warren DK, Guth RM, Coopersmith CM, Merz LR, Zack JE, Fraser VJ. 2006. Epidemiology of methicillin-resistant Staphylococcus aureus colonization in a surgical intensive care unit. Infect Control Hosp Epidemiol 27:1032–1040. doi:10.1086/507919. 17006809

[14] Pineles L, Morgan DJ, Lydecker A, Johnson JK, Sorkin JD, Langenberg P, Blanco N, Lesse A, Sellick J, Gupta K, Leykum L, Cadena J, Lepcha N, Roghmann M-C. 2017. Transmission of methicillin-resistant Staphylococcus aureus to healthcare worker gowns and gloves during care of residents in Veterans Affairs nursing homes. Am J Infect Control 45:947–953. doi:10.1016/j.ajic.2017.03.004. 28431853 PMC6453115

[15] Jackson SS, Harris AD, Magder LS, et al. 2018. Bacterial burden is associated with increased transmission to healthcare workers from patients colonized with vancomycin-resistant Enterococcus. Am J Infect Control 47:13–17. 30268592 10.1016/j.ajic.2018.07.011 PMC6452858

[16] Lee AS, De Lencastre H, Garau J, et al. 2018. Methicillin-resistant Staphylococcus aureus. Nat Rev Dis Prim 4:18034. 29849094 10.1038/nrdp.2018.33

[17] Haddad O, Merghni A, Elargoubi A, Rhim H, Kadri Y, Mastouri M. 2018. Comparative study of virulence factors among methicillin resistant Staphylococcus aureus clinical isolates. BMC Infect Dis 18:560. doi:10.1186/s12879-018-3457-2. 30424731 PMC6234561

[18] Watkins RR, David MZ, Salata RA. 2012. Current concepts on the virulence mechanisms of meticillin-resistant Staphylococcus aureus. J Med Microbiol 61:1179–1193. doi:10.1099/jmm.0.043513-0. 22745137 PMC4080747

[19] Donlan RM. 2002. Biofilms: microbial life on surfaces. Emerg Infect Dis 8:881–890. doi:10.3201/eid0809.020063. 12194761 PMC2732559

[20] Jain A, Gupta Y, Agrawal R, Khare P, Jain SK. 2007. Biofilms - a microbial life perspective: a critical review. Crit Rev Ther Drug Carrier Syst 24:393–443. doi:10.1615/critrevtherdrugcarriersyst.v24.i5.10. 18197780

[21] Schroeder K, Jularic M, Horsburgh SM, Hirschhausen N, Neumann C, Bertling A, Schulte A, Foster S, Kehrel BE, Peters G, Heilmann C. 2009. Molecular characterization of a novel Staphylococcus aureus surface protein (SasC) involved in cell aggregation and biofilm accumulation. PLoS One 4:e7567. doi:10.1371/journal.pone.0007567. 19851500 PMC2761602

[22] Foster TJ, Geoghegan JA, Ganesh VK, Höök M. 2014. Adhesion, invasion and evasion: the many functions of the surface proteins of Staphylococcus aureus. Nat Rev Microbiol 12:49–62. doi:10.1038/nrmicro3161. 24336184 PMC5708296

[23] Sahl JW, Beckstrom-Sternberg SM, Babic-Sternberg JS, et al. 2015. The In Silico Genotyper (ISG): an open-source pipeline to rapidly identify and annotate nucleotide variants for comparative genomics applications. bioRxiv 10.1101/015578.

[24] Sahl JW, Johnson JK, Harris AD, Phillippy AM, Hsiao WW, Thom KA, Rasko DA. 2011. Genomic comparison of multi-drug resistant invasive and colonizing Acinetobacter baumannii isolated from diverse human body sites reveals genomic plasticity. BMC Genomics 12:291. doi:10.1186/1471-2164-12-291. 21639920 PMC3126785

[25] Strommenger B, Braulke C, Heuck D, Schmidt C, Pasemann B, Nübel U, Witte W. 2008. spa typing of Staphylococcus aureus as a frontline tool in epidemiological typing. J Clin Microbiol 46:574–581. doi:10.1128/JCM.01599-07. 18032612 PMC2238071

[26] Bartels MD, Petersen A, Worning P, Nielsen JB, Larner-Svensson H, Johansen HK, Andersen LP, Jarløv JO, Boye K, Larsen AR, Westh H. 2014. Comparing whole-genome sequencing with sanger sequencing for spa typing of methicillin-resistant staphylococcus aureus. J Clin Microbiol 52:4305–4308. doi:10.1128/JCM.01979-14. 25297335 PMC4313303

[27] Kaya H, Hasman H, Larsen J, Stegger M, Johannesen TB, Allesøe RL, Lemvigh CK, Aarestrup FM, Lund O, Larsen AR. 2018. SCCmecFinder, a web-based tool for typing of Staphylococcal cassette chromosome mec in Staphylococcus aureus using whole-genome sequence data. mSphere 3:e00612-17. doi:10.1128/mSphere.00612-17. 29468193 PMC5812897

[28] Enright MC, Day NP, Davies CE, Peacock SJ, Spratt BG. 2000. Multilocus sequence typing for characterization of methicillin-resistant and methicillin-susceptible clones of Staphylococcus aureus. J Clin Microbiol 38:1008–1015. doi:10.1128/JCM.38.3.1008-1015.2000. 10698988 PMC86325

[29] Geoghegan JA, Corrigan RM, Gruszka DT, Speziale P, O'Gara JP, Potts JR, Foster TJ. 2010. Role of surface protein SasG in biofilm formation by Staphylococcus aureus. J Bacteriol 192:5663–5673. doi:10.1128/JB.00628-10. 20817770 PMC2953683

[30] Belyi Y, Rybolovlev I, Polyakov N, Chernikova A, Tabakova I, Gintsburg A. 2018. Staphylococcus aureus surface protein G is an immunodominant protein and a possible target in an anti-biofilm drug development. Open Microbiol J 12:94–106. doi:10.2174/1874285801812010094. 29785216 PMC5944129

[31] Mazmanian SK, Liu G, Ton-That H, Schneewind O. 1999. Staphylococcus aureus sortase, an enzyme that anchors surface proteins to the cell wall. Science 285:760–763. doi:10.1126/science.285.5428.760. 10427003

[32] Perry AM, Ton-That H, Mazmanian SK, Schneewind O. 2002. Anchoring of surface proteins to the cell wall of Staphylococcus aureus. III. Lipid II is an in vivo peptidoglycan substrate for sortase-catalyzed surface protein anchoring. J Biol Chem 277:16241–16248. doi:10.1074/jbc.M109194200. 11856734

[33] Siegel SD, Reardon ME, Ton-That H. 2017. Anchoring of LPXTG-like proteins to the gram-positive cell wall envelope. Current Topics in Microbiology and Immunology 404:159–175. doi:10.1007/82_2016_8. 27097813

[34] Hiron A, Falord M, Valle J, Débarbouillé M, Msadek T. 2011. Bacitracin and nisin resistance in Staphylococcus aureus: a novel pathway involving the BraS/BraR two-component system (SA2417/SA2418) and both the BraD/BraE and VraD/VraE ABC transporters. Mol Microbiol 81:602–622. doi:10.1111/j.1365-2958.2011.07735.x. 21696458

[35] Yoshida Y, Matsuo M, Oogai Y, Kato F, Nakamura N, Sugai M, Komatsuzawa H. 2011. Bacitracin sensing and resistance in Staphylococcus aureus. FEMS Microbiol Lett 320:33–39. doi:10.1111/j.1574-6968.2011.02291.x. 21517944

[36] Han CG, Shiga Y, Tobe T, Sasakawa C, Ohtsubo E. 2001. Structural and functional characterization of IS679 and IS66-family elements. J Bacteriol 183:4296–4304. doi:10.1128/JB.183.14.4296-4304.2001. 11418571 PMC95320

[37] Diep BA, Gill SR, Chang RF, Phan TH, Chen JH, Davidson MG, Lin F, Lin J, Carleton HA, Mongodin EF, Sensabaugh GF, Perdreau-Remington F. 2006. Complete genome sequence of USA300, an epidemic clone of community-acquired meticillin-resistant Staphylococcus aureus. Lancet 367:731–739. doi:10.1016/S0140-6736(06)68231-7. 16517273

[38] Peacock SJ, Moore CE, Justice A, Kantzanou M, Story L, Mackie K, O'Neill G, Day NPJ. 2002. Virulent combinations of adhesin and toxin genes in natural populations of Staphylococcus aureus. Infect Immun 70:4987–4996. doi:10.1128/IAI.70.9.4987-4996.2002. 12183545 PMC128268

[39] Holden MTG, Feil EJ, Lindsay JA, Peacock SJ, Day NPJ, Enright MC, Foster TJ, Moore CE, Hurst L, Atkin R, Barron A, Bason N, Bentley SD, Chillingworth C, Chillingworth T, Churcher C, Clark L, Corton C, Cronin A, Doggett J, Dowd L, Feltwell T, Hance Z, Harris B, Hauser H, Holroyd S, Jagels K, James KD, Lennard N, Line A, Mayes R, Moule S, Mungall K, Ormond D, Quail MA, Rabbinowitsch E, Rutherford K, Sanders M, Sharp S, Simmonds M, Stevens K, Whitehead S, Barrell BG, Spratt BG, Parkhill J. 2004. Complete genomes of two clinical Staphylococcus aureus strains: evidence for the evolution of virulence and drug resistance. Proc Natl Acad Sci USA 101:9786–9791. doi:10.1073/pnas.0402521101. 15213324 PMC470752

[40] Baba T, Takeuchi F, Kuroda M, Yuzawa H, Aoki K-i, Oguchi A, Nagai Y, Iwama N, Asano K, Naimi T, Kuroda H, Cui L, Yamamoto K, Hiramatsu K. 2002. Genome and virulence determinants of high virulence community-acquired MRSA. Lancet 359:1819–1827. doi:10.1016/s0140-6736(02)08713-5. 12044378

[41] Kuroda M, Ohta T, Uchiyama I, Baba T, Yuzawa H, Kobayashi I, Cui L, Oguchi A, Aoki K, Nagai Y, Lian J, Ito T, Kanamori M, Matsumaru H, Maruyama A, Murakami H, Hosoyama A, Mizutani-Ui Y, Takahashi NK, Sawano T, Inoue R, Kaito C, Sekimizu K, Hirakawa H, Kuhara S, Goto S, Yabuzaki J, Kanehisa M, Yamashita A, Oshima K, Furuya K, Yoshino C, Shiba T, Hattori M, Ogasawara N, Hayashi H, Hiramatsu K. 2001. Whole genome sequencing of methicillin-resistant Staphylococcus aureus. Lancet 357:1225–1240. doi:10.1016/s0140-6736(00)04403-2. 11418146

[42] Argudín MÁ, Mendoza MC, Rodicio MR. 2010. Food poisoning and Staphylococcus aureus enterotoxins. Toxins (Basel) 2:1751–1773. doi:10.3390/toxins2071751. 22069659 PMC3153270

[43] Ando E, Monden K, Mitsuhata R, Kariyama R, Kumon H. 2004. Biofilm formation among methicillin-resistant Staphylococcus aureus isolates from patients with urinary tract infection. Acta Med Okayama 58:207–214. 15551758 10.18926/AMO/32090

[44] McClure JA, Zhang K. 2017. Complete genome sequence of the methicillin-resistant Staphylococcus aureus colonizing strain M92. Genome Announc 5:e00478-17. doi:10.1128/genomeA.00478-17. 28596402 PMC5465621

[45] Bardy JJ, Sarovich DS, Price EP, Steinig E, Tong S, Drilling A, Ou J, Vreugde S, Wormald P-J, Psaltis AJ. 2018. Staphylococcus aureus from patients with chronic rhinosinusitis show minimal genetic association between polyp and non-polyp phenotypes. BMC Ear Nose Throat Disord 18:16. doi:10.1186/s12901-018-0064-1. 30349419 PMC6192324

[46] Kennedy AD, Otto M, Braughton KR, Whitney AR, Chen L, Mathema B, Mediavilla JR, Byrne KA, Parkins LD, Tenover FC, Kreiswirth BN, Musser JM, DeLeo FR. 2008. Epidemic community-associated methicillin-resistant Staphylococcus aureus: recent clonal expansion and diversification. Proc Natl Acad Sci USA 105:1327–1332. doi:10.1073/pnas.0710217105. 18216255 PMC2234137

[47] Asadollahi P, Farahani NN, Mirzaii M, Khoramrooz SS, van Belkum A, Asadollahi K, Dadashi M, Darban-Sarokhalil D. 2018. Distribution of the most prevalent spa types among clinical isolates of methicillin-resistant and -susceptible Staphylococcus aureus around the world: a review. Front Microbiol 9:163. doi:10.3389/fmicb.2018.00163. 29487578 PMC5816571

[48] McClure J-AM, Lakhundi S, Kashif A, Conly JM, Zhang K. 2018. Genomic comparison of highly virulent, moderately virulent, and avirulent strains from a genetically closely-related MRSA ST239 sub-lineage provides insights into pathogenesis. Front Microbiol 9:1531. doi:10.3389/fmicb.2018.01531. 30042755 PMC6048232

[49] Adediran T, Hitchcock S, O’Hara LM, Michalski JM, Johnson JK, Calfee DP, Miller LG, Hazen TH, Rasko DA, Harris AD. 2019. Examination of 388 Staphylococcus aureus isolates from intensive care unit patients. Microbiol Resour Announc 8:e01246-19. doi:10.1128/MRA.01246-19. 31727718 PMC6856284

[50] Bolger AM, Lohse M, Usadel B. 2014. Trimmomatic: a flexible trimmer for Illumina sequence data. Bioinformatics 30:2114–2120. doi:10.1093/bioinformatics/btu170. 24695404 PMC4103590

[51] Bankevich A, Nurk S, Antipov D, Gurevich AA, Dvorkin M, Kulikov AS, Lesin VM, Nikolenko SI, Pham S, Prjibelski AD, Pyshkin AV, Sirotkin AV, Vyahhi N, Tesler G, Alekseyev MA, Pevzner PA. 2012. SPAdes: a new genome assembly algorithm and its applications to single-cell sequencing. J Comput Biol 19:455–477. doi:10.1089/cmb.2012.0021. 22506599 PMC3342519

[52] Sahl JW, Gregory Caporaso J, Rasko DA, Keim P. 2014. The large-scale blast score ratio (LS-BSR) pipeline: a method to rapidly compare genetic content between bacterial genomes. PeerJ 2:e332. doi:10.7717/peerj.332. 24749011 PMC3976120

[53] Gu Z, Eils R, Schlesner M. 2016. Complex heatmaps reveal patterns and correlations in multidimensional genomic data. Bioinformatics 32:2847–2849. doi:10.1093/bioinformatics/btw313. 27207943

[54] R Core Team. R: a language and environment for statistical computing. 2018. https://www.gbif.org/tool/81287/r-a-language-and-environment-for-statistical-computing. Accessed June 24, 2020.

[55] Brynildsrud O, Bohlin J, Scheffer L, Eldholm V. 2016. Rapid scoring of genes in microbial pan-genome-wide association studies with Scoary. Genome Biol 17:238. doi:10.1186/s13059-016-1108-8. 27887642 PMC5124306

[56] Jolley KA, Bray JE, Maiden MCJ. 2018. Open-access bacterial population genomics: BIGSdb software, the PubMLST.org website and their applications. Wellcome Open Res 3:124. doi:10.12688/wellcomeopenres.14826.1. 30345391 PMC6192448

[57] Ito T, Hiramatsu K, Oliveira DC. 2009. Classification of staphylococcal cassette chromosome mec (SCCmec): guidelines for reporting novel SCCmec elements. Antimicrob Agents Chemother 53:4961–4967. 19721075 10.1128/AAC.00579-09 PMC2786320

[58] Kondo Y, Ito T, Ma XX, Watanabe S, Kreiswirth BN, Etienne J, Hiramatsu K. 2007. Combination of multiplex PCRs for staphylococcal cassette chromosome mec type assignment: rapid identification system for mec, ccr, and major differences in junkyard regions. Antimicrob Agents Chemother 51:264–274. doi:10.1128/AAC.00165-06. 17043114 PMC1797693

[59] Camacho C, Coulouris G, Avagyan V, Ma N, Papadopoulos J, Bealer K, Madden TL. 2009. BLAST+: architecture and applications. BMC Bioinformatics 10:421. doi:10.1186/1471-2105-10-421. 20003500 PMC2803857

[60] Stamatakis A. 2014. RAxML version 8: a tool for phylogenetic analysis and post-analysis of large phylogenies. Bioinformatics 30:1312–1313. doi:10.1093/bioinformatics/btu033. 24451623 PMC3998144

