# Erratum for Adediran et al., “Comparative Genomics Identifies Features Associated with Methicillin-Resistant Staphylococcus aureus (MRSA) Transmission in Hospital Settings”

**DOI:** 10.1128/msphere.00342-22

**Published:** 2022-08-24

**Authors:** Timileyin Adediran, Stephanie Hitchcock, Lyndsay M. O’Hara, Jane M. Michalski, J. Kristie Johnson, David P. Calfee, Loren G. Miller, Tracy H. Hazen, Anthony D. Harris, David A. Rasko

**Affiliations:** a Institute for Genome Sciences, University of Maryland School of Medicine, Baltimore, Maryland, USA; b Department of Microbiology and Immunology, University of Maryland School of Medicine, Baltimore, Maryland, USA; c Department of Pathology, University of Maryland School of Medicine, Baltimore, Maryland, USA; d Division of Infectious Diseases, Weill Cornell Medicine, New York, New York, USA; e Lundquist Institute for Biomedical Innovation at Harbor-UCLA Medical Center, Torrance, California, USA; f Department of Epidemiology and Public Health, University of Maryland School of Medicine, Baltimore, Maryland, USA

## ERRATUM

Volume 7, no. 3, e00116-22, 2022, https://doi.org/10.1128/msphere.00116-22. Page 1: This article was published on 17 May 2022 with Timileyin Adediran’s surname misspelled as “Adedrian” in the byline. The corrected byline is shown above.

