# Supplementary figures and images for: Comparative Genomics Identifies Features Associated with Methicillin-Resistant Staphylococcus aureus (MRSA) Transmission in Hospital Settings
Source: mSphere. 2022 May 17;7(3):e00116-22. doi: 10.1128/msphere.00116-22 (PMC9241550; doi:10.1128/msphere.00116-22)

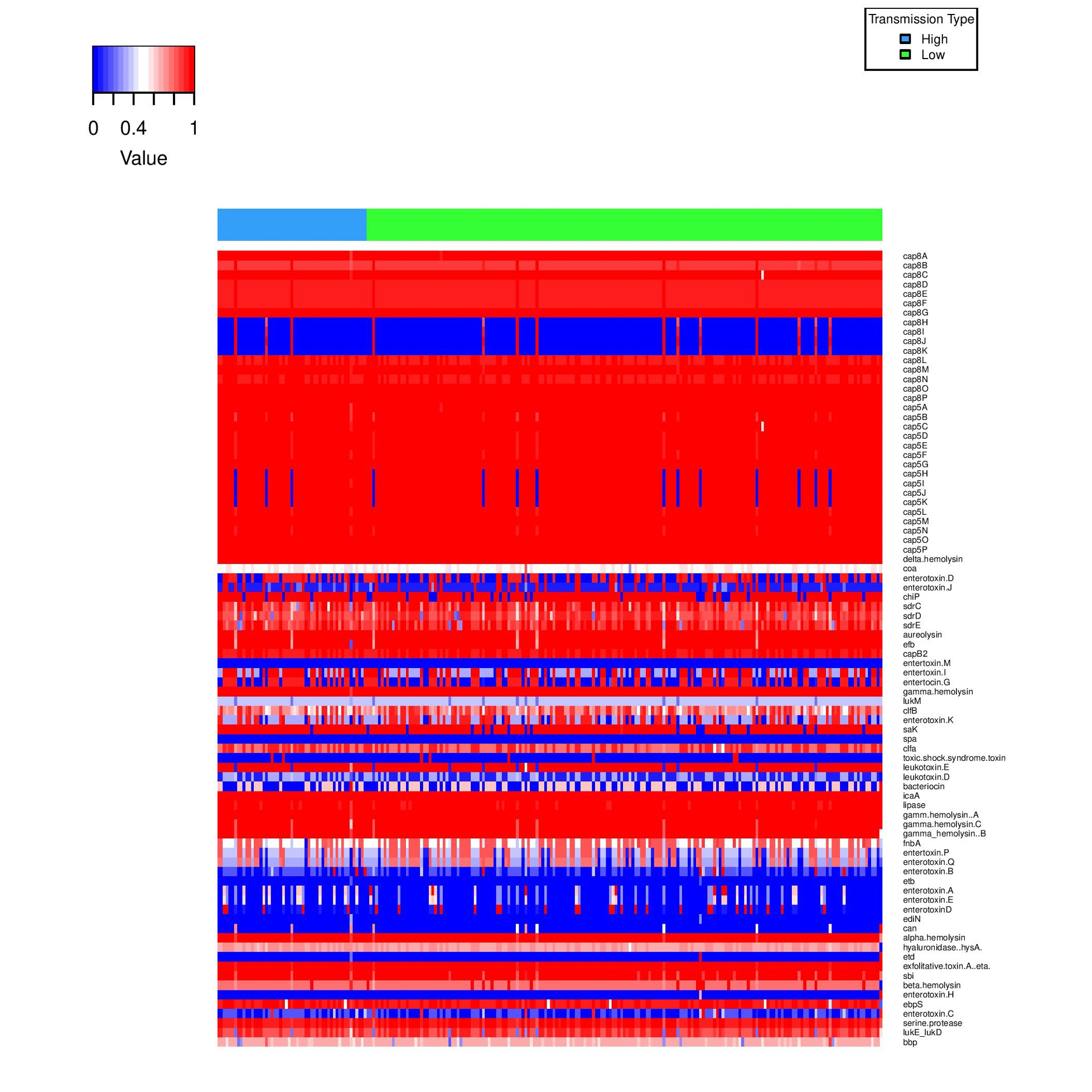

Supplement: FIG S1 [file msphere.00116-22-s0006.tif]
